# Numerical simulation analysis of carbon defects in the buffer on vertical leakage and breakdown of GaN on silicon epitaxial layers

**DOI:** 10.1038/s41598-023-41678-1

**Published:** 2023-09-08

**Authors:** Weicheng Cao, Chunyan Song, Hui Liao, Ningxuan Yang, Rui Wang, Guanghui Tang, Hongyu Ji

**Affiliations:** 1 Department of Physics, College of Sciences, Shihezi University, Shihezi, 832000 China; 2 Xinjiang Production & Construction Corps Key Laboratory of Advanced Energy Storage Materials and Technology, Shihezi University, Shihezi, 832000 China

**Keywords:** Semiconductors, Condensed-matter physics

## Abstract

Carbon doping in GaN-on-Silicon (Si) epitaxial layers is an essential way to reduce leakage current and improve breakdown voltage. However, complicated occupy forms caused by carbon lead to hard analysis leakage/breakdown mechanisms of GaN-on-Si epitaxial layers. In this paper, we demonstrate the space charge distribution and intensity in GaN-on-Si epitaxial layers from 0 to 448 V by simulation. Depending on further monitoring of the trapped charge density of C_N_ and C_Ga_ in carbon-doped GaN at 0.1 μm, 0.2 μm, 1.8 μm and 1.9 μm from unintentionally doped GaN/carbon-doped GaN interface, we discuss the relationship between space charge and plateau, breakdown at C_N_ concentrations from 6 × 10^16^ cm^−3^ to 6 × 10^18^ cm^−3^. The results show that C_N_ in different positions of carbon-doped GaN exhibits significantly different capture and release behaviors. By utilizing the capture and release behavior differences of C_N_ at different positions in carbon-doped GaN, the blocking effect of space charge at unintentionally doped GaN/carbon-doped GaN interface on electron conduction was demonstrated. The study would help to understand the behavior of C_N_ and C_Ga_ in GaN-on-Si epitaxial layers and more accurate control of C_N_ and C_Ga_ concentration at different positions in carbon-doped GaN to improve GaN-on-Si device performance.

Metal–organic Chemical Vapor Deposition (MOCVD) has become the most common growth method for gallium nitride (GaN) owing to its advantages of easy control, high crystal quality, and relatively simple equipment, which is conducive to large-scale industrialization^1,2^. However, MOCVD will inevitably introduce carbon impurities leading to undesired leakage paths in the growth of GaN-based epitaxial layers^3^. Carbon is also often intentionally doped in GaN to obtain the high resistance GaN region, which is important for high frequency, high power and high mobility transistors with semi-insulating or insulating properties^4^. Nevertheless, carbon can occupy the N site to form C_N_ defect, the Ga site to form C_Ga_ defect, or compounds such as C_N_–O_N_, C_N_–H_i_, and other forms in GaN epitaxial layers^5^, which would lead to complex defect formation problems. In addition, carbon defects would bring issues such as current collapse, which lead to excessive power loss and device efficiency reduced^6,7^.

Many studies have been done on carbon-doped GaN to solve the above problems^4,8^. Researches have shown that C_N_ is the predominant defect type that results in deep traps with an energy level of E_V_ + (0.86–0.9) eV^5,9^, and no associated compound impurities are formed at low carbon doping concentrations^10^. However, C_Ga_ always accompanied by C_N_ causes the self-compensation effect occurs, which makes the C_N_ concentration much lower than the doping concentration and thus reduces the device performance^11^. Studies have revealed that the ratio of concomitant donor defects to acceptor defects is roughly 0.5 in carbon-doped GaN^12^. The sum of the acceptor concentration and the donor concentration determines the breakdown voltage, and the effective defect concentration determines the current-collapse magnitude^11^. The reduction of leakage current and the increase of breakdown voltage by the introduction of carbon doping are dependent on the charging/discharging process of the carbon defects.

However, the concentration and dynamic behavior of the carbon defects vary significantly with the total carbon doping concentration and growth conditions^10^. Even at the same carbon doping concentration significant variations in C_N_ and C_Ga_ concentration can occur^3,13^. Therefore, the possible effects of variations in the concentration of carbon defects need to be analyzed under precise control of other carbon defects concentration. Furthermore, due to the complicated carbon occupy forms, the discussion of carbon defects is usually based on the total carbon doping concentrations or simple one-dimensional analysis^14,15^, rather than the specific defect concentration or directly observing the trap state, which has resulted in other possible effects caused by carbon defects not being taken into account. It also needs to be further clarified whether the space charge formed by carbon defects through charging/discharging affects the leakage^16^.

In this work, we use Sentaurus TCAD simulation to discuss the role of the specific energy level of C_N_ and C_Ga_ defects to analyze their influence on vertical leakage/breakdown at different concentrations in GaN-on-Si epitaxial layers. The relationship between space charge and leakage/breakdown is analyzed intuitively with the advantage of simulation. The roles of C_N_ and C_Ga_ in the whole leakage process were elucidated by monitoring the trapped charge density. Understanding the complex dynamic mechanisms of acceptor and donor traps in carbon-doped GaN is great significance for guiding the improvement of GaN device performance^14^.

This paper is organized as follows, Section Modeling details the relevant settings for the simulation of GaN-on-Si epitaxial layers. Section Result and Discussion shows vertical leakage characteristics at different C_N_ concentrations at first. Followed by a discussion of the variation of space charge with different voltage, and the variation of C_N_ and C_Ga_ trapping in GaN:C. Section Result and Discussion-Plateau discusses the effect of C_N_ and C_Ga_ on the plateau in vertical leakage characteristics by space charge, and Section Result and Discussion-Breakdown discuss the effect of C_N_ and C_Ga_ on breakdown via space charge. Finally, the breakdown mechanism of the additional introduction of C_N_ on top of GaN:C to increase the breakdown voltage was confirmed. In the log J-V diagram of leakage characterization, the region of current-limiting growth is called the plateau region, the point of sudden rapid current growth is called the kink, and the current of 1 A/cm^2^ is defined as breakdown.

## Modeling

**Figure 1:**
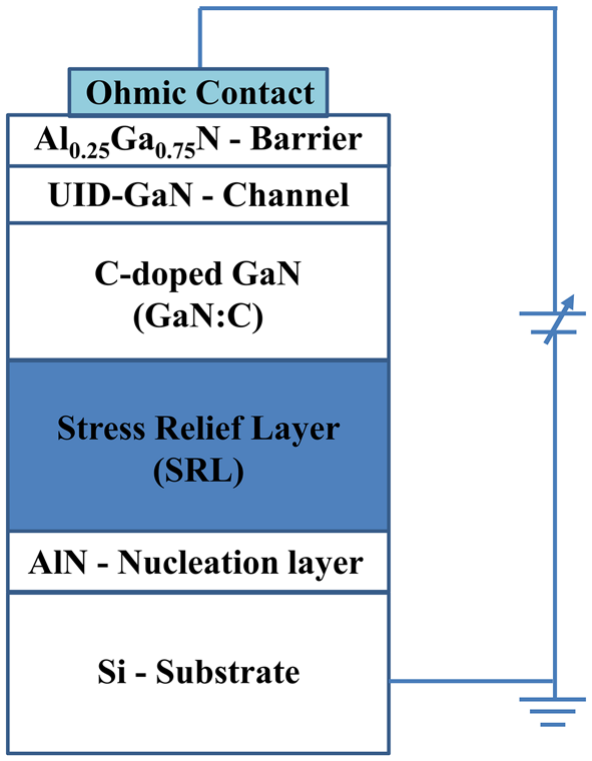
Structure of the GaN-on-Si constructed by simulation.

In this paper, Sentaurus TCAD was used for 2D simulation of electrical properties of GaN-on-Si epitaxial layers. The structure is shown in Fig. 1. From bottom to top, consists of silicon substrate, 300 nm AlN layer, 1.2 μm step-graded AlGaN stress relief layers (SRL), common ratio combinations of 75%, 50% and 25% have been chosen for the SRL component ratios^8^, 2 µm carbon-doped GaN (GaN:C) layer, 200 nm unintentionally doped GaN (UID-GaN) layer, and 25 nm Al_0.25_Ga_0.75_N barrier layer. The common carbon doping concentration of 1 × 10^19^ cm^−3^ was set in the GaN:C layer^4^. Except for the Al_0.25_Ga_0.75_N barrier layer, background carbon doping of 1 × 10^15^ cm^−3^ was considered for all nitride layers^11^.

Based on the measured results, E_V_ + 0.9 eV was chosen as the energy level of C_N_ defects and E_C_—0.11 eV was chosen as the energy level of C_Ga_ defects^11^ 1 × 10^–15^ cm^2^ was selected as the electron and hole capture cross-section size for C_N_ and C_Ga_.^17^ Referring to the results of Refs.^18,19^, the C_N_ concentration 6 × 10^16^ cm^−3^, 4 × 10^17^ cm^−3^, 6 × 10^17^ cm^−3^ and 6 × 10^18^ cm^−3^ were introduced in 1 × 10^19^ cm^−3^ carbon concentration GaN:C layer, and the C_Ga_ concentration has been set as 50% of the corresponding C_N_ defect concentration^11,12^. The following descriptions of C_N_ were carried out under a fixed 50% ratio of C_Ga_.

Concerning dislocations and impurities, the defect energy level of 0.6 eV and 1.3 eV with a concentration of 5 × 10^16^ cm^−3^ are introduced in the AlN layer and SRL^20,21^.

To simulate trap effect on GaN-on-Si epitaxial layers, both Shockley–Read–Hall (SRH) and Poole Frankel (PF) conduction mechanisms are introduced in the defect-containing region^16,22^. The band-to-band model^23^, thermionic emission mechanism^18,24^, and trap-assisted tunneling (TAT) model are introduced to simulate the current conduction process^18,25^. Impact ionization based on Chynoweth's law is taken into account in the simulation, which *a* (electrons) is 2.32 × 10^6^ cm^−1^, *b* (electrons) is 1.4 × 10^7^ V/cm, *a* (holes) is 5.41 × 10^6^ cm^−1^ and *b* (holes) is 1.89 × 10^7^ V/cm^11^.

For the accuracy of the simulation, the mesh within the AlN layer and at the AlN/Si interface has been specifically refined to accurately simulate the complexities of the electron channels here^26^.

## Results and discussion

**Figure 2:**
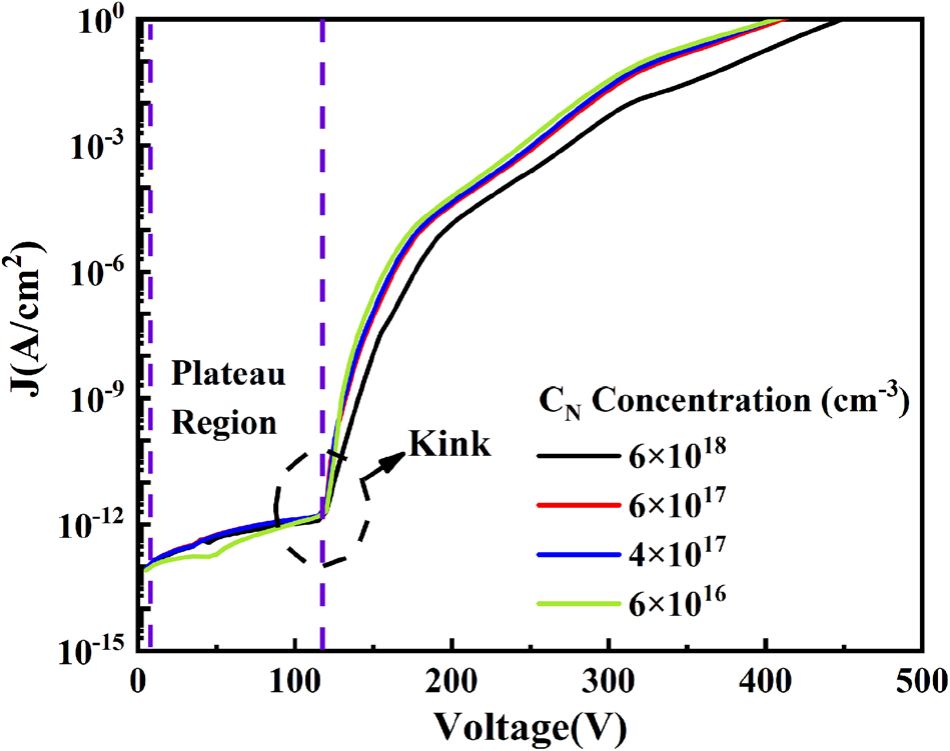
The log J-V curve with the concentration of C_N_ from 6 × 10^16^ cm^−3^, 4 × 10^17^ cm^−3^, 6 × 10^17^ cm^−3^ and 6 × 10^18^ cm^−3^, the concentration of C_Ga_ is 50% of each C_N_ above in GaN:C layer.

To investigate the effect of C_N_ concentration on the leakage characteristics of GaN-on-Si epitaxial layers, the log J–V characteristic for C_N_ concentration from 6 × 10^16^ cm^−3^ to 6 × 10^18^ cm^−3^ is shown in Fig. 2. As C_N_ increases from 6 × 10^16^ cm^−3^ to 6 × 10^18^ cm^−3^, the breakdown voltage increases from 378 to 448 V. Kinks at about 120 V changed a little with the increase of C_N_ concentration. The result of the leakage characteristics here is consistent with the actual situation compared with the results of Refs.^11,27^. Since C_N_ and C_Ga_ through the charging/discharging process will result the change of space charge^14^. Therefore, it is a good choice to analyze the total effect of C_N_ and C_Ga_ charging/discharging process by observing the change in space charge.

**Figure 3:**
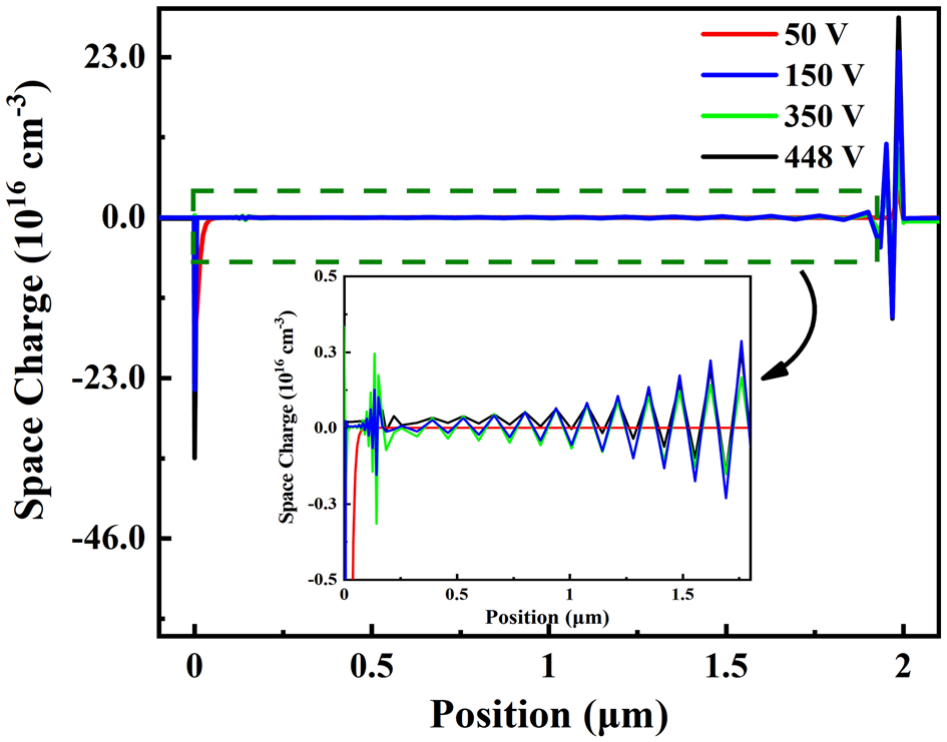
Space charge diagram at voltages 50 V, 150 V, 350 V, 448 V with C_N_ 6 × 10^18^ cm^−3^ in GaN:C layer.

To further investigate the influence of space charge on the electron conduction process and how C_N_ and C_Ga_ increase breakdown voltage in GaN-on-Si epitaxial layers. The space charge for GaN:C at 50 V, 150 V, 350 V and 448 V for C_N_ 6 × 10^18^ cm^−3^ are shown in Fig. 3, which were derived by intercepting the space charge simulated data at middle positions of GaN-on-Si epitaxial layers. UID-GaN/GaN:C interface was set at 0 μm and at 2 μm was set as GaN:C/SRL interface. The simulation results are consistent with Refs.^14,19^. As shown in Fig. 3, it was found the following:

(i) High density of space charge appears at 0 μm and 2 μm. This is due to the difference in conductivity between the different layers^13^.
(ii) Alternating positive and negative space charges appear within GaN:C at 150 V, 350 V and 448 V. By the condition of electrical neutrality, space charges of opposite electrical properties are bound to appear on either side of the space charge at the interface, and this opposite space charge further induces space charge in the adjacent region. Therefore, the GaN:C layer, by virtue of it’s larger thickness, appears to have alternating low-density space charges.
(iii) At 448 V, contiguous positive space charge appears in the middle of GaN:C and stronger negative space charge appears at 0 μm. At higher voltages, the high electric field increases the number of carriers captured by C_N_ and C_Ga_.

However, positive space charge appears in GaN:C layer, which is not consistent with the trap state after C_N_ capture electrons captures suggests that the C_N_ in GaN:C layer may not always capture electrons, but release electrons to make the appearance of positive space charge. If so, the release of electrons by C_N_ cannot explain the decrease in plateau current caused by the increase in C_N_ concentration in Fig. 2, suggesting that the C_N_ and C_Ga_ trapping process is needed to discuss further.

**Figure 4:**
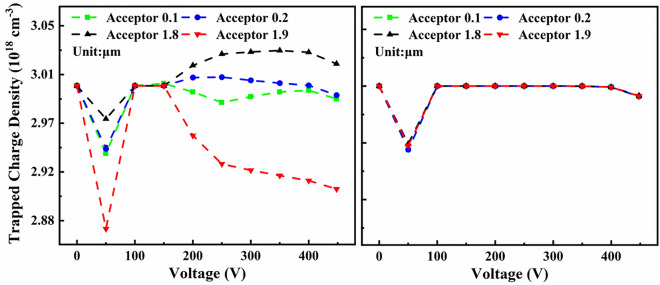
Trapped charge density–Voltage plot of (left) C_N_, (right) C_Ga_ at 0.1 μm, 0.2 μm, 1.8 μm and 1.9 μm in GaN:C layer from UID-GaN/GaN: C interface.

To further investigate the trap states behavior of C_N_ and C_Ga_ at different voltages, we monitored the trap states of C_N_ and C_Ga_ at 0 V to 448 V at the distance 0.1 μm, 0.2 μm, 1.8 μm, 1.9 μm from UID-GaN/ GaN:C interface to GaN:C layer, which shown in Fig. 4. For C_N_ in Fig. 4, it was found that:

(i) The trapped charge in GaN:C at 0.1 μm, 0.2 μm, 1.8 μm and 1.9 μm from the GaN:C/UID-GaN interface decrease from 0 to 50 V. Because the plateau region has been entered at 50 V, the defects within the GaN:C continue to capture electrons and the region of space charge caused by polarization begins to expand^18^. To satisfy the electrically neutral condition, the neighboring locations of the interface keep releasing charge.
(ii) The trapped charge of C_N_ at 0.1 μm, 0.2 μm, 1.8 μm and 1.9 μm from the UID-GaN/ GaN:C interface tends to increase from 50 to150V. As the voltage increases, the number of electrons entering the GaN increases making the number of captures increase.
(iii) The trapped charge of C_N_ at 1.8 μm and 0.2 μm from the GaN:C/UID-GaN interface increase then decrease after 150 V. For C_N_ at 0.1 μm from the GaN:C/UID-GaN interface, trapped charge decline and then grow after 150 V. For C_N_ at 1.9 μm, trapped charge decreased from 150 to 448 V. The reason for the difference between the different locations is that electrical properties space charge on both sides is disparate.

For C_Ga_ shown in Fig. 4, the trapped charge of C_Ga_ decline from 0 to 50 V, then increase from 50 to 100 V. To satisfy electrically neutral conditions, C_Ga_ releasing charge in response the captured electrons at the interface of GaN:C and the expansion of space charge regions. Between 100 and 400 V, the trapped charge of C_Ga_ is essentially constant. For the C_Ga_ shallow energy level, it always maintains complete ionization^11^. Therefore, C_Ga_ trapped charge is essentially constant. Finally, under the further expansion of the space charge region, decreases significantly between 400 and 448 V.

### Plateau

Since the existence of the barrier at AlN/Si interface, many electrons are confined near the AlN/Si interface at low voltages^18^. Even if few electrons tunnel through the barrier^24^, they are trapped by defects in the AlN layer, SRL, or GaN:C layer, causing the current to increase slowly and then forming a plateau. When the applied voltage is high enough, lots of electrons at the AlN/Si interface would pass through the barrier by thermionic emission^18^. The kink in the log J-V curve would appear until the defects in the GaN:C layer were full filled^27^.

In brief, in the plateau region, C_N_ plays a major role in forming space charge by charging/discharging, whereas the C_Ga_ capture charge remains essentially constant. The increase of C_N_ helps to capture more electrons, thus causing the plateau current drop. Since the reduction of the plateau current requires C_N_ to capture electrons, the study on the capture and release behavior of C_N_ at different positions indicates that the plateau current can be better reduced by introducing more C_N_ at the C_N_ capture position.

### Breakdown

To further discuss the effect of C_N_ and C_Ga_ on breakdown, we investigate the change near breakdown in Fig. 2 and explain the reasons for the change in conjunction with the results in Figs. 3 and 4. Firstly, in Fig. 2, the slope of log J–V starts to decrease at C_N_ 6 × 10^16^ cm^−3^–6 × 10^17^ cm^−3^ at about 330 V. When C_N_ increases to 6 × 10^18^ cm^−3^, the slope decreases at about 310 V, and the breakdown voltage rises from 378 to 448 V with the increase in C_N_ concentration. Secondly, in Fig. 3, it is found that when the voltage reaches 350 V, a contiguous region of positive space charge appears in GaN:C layer, while a stronger negative space charge appears in UID-GaN layer. Finally, Fig. 4 shows that the C_N_ trapped charge at 400 V, it starts to decrease at 0.1 μm, 0.2 μm, 1.8 μm and 1.9 μm in GaN:C from the GaN:C/UID-GaN interface.

**Figure 5:**
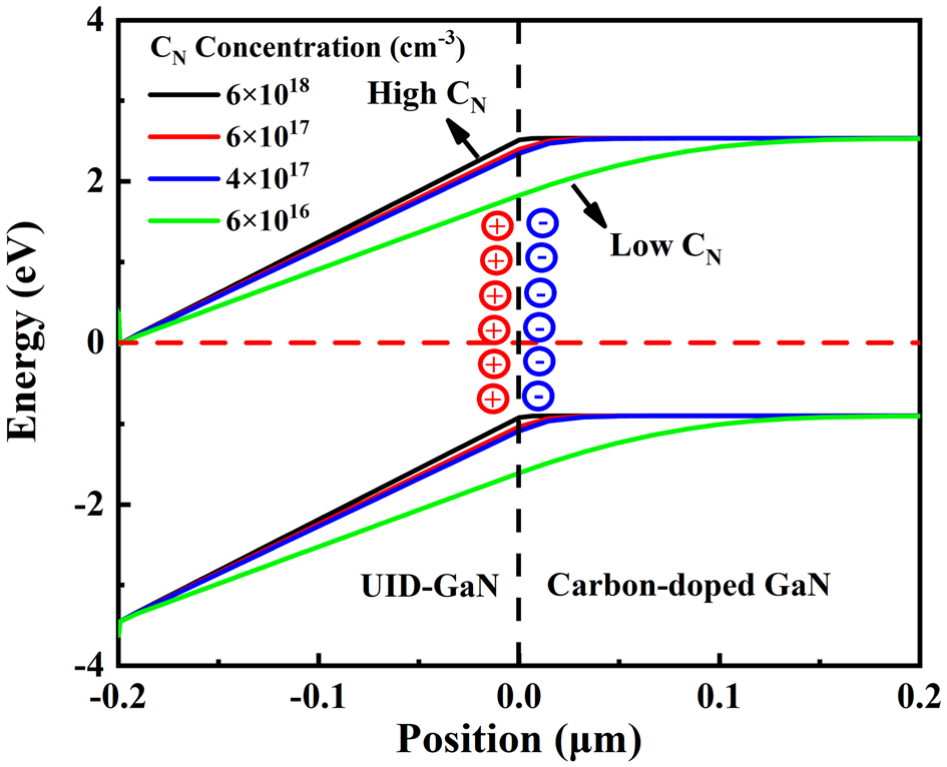
Energy band of the GaN-on-Si constructed at C_N_ concentration of 6 × 10^16^ cm^−3^, 4 × 10^17^ cm^−3^, 6 × 10^17^ cm^−3^ and 6 × 10^18^ cm^−3^.

Due to the Maxwell–Wagner effect, there is a negative space charge region at the UID-GaN/GaN:C interface that increases with C_N_ concentration^13^. High C_N_ concentrations cause sufficiently narrow negative space charge region within GaN:C, while lower C_N_ causes this space charge region expand into GaN:C. This is reflected in the energy band as a bending of the energy band (Fig. 5). As the electrical stress increases, the ionization of C_N_ leads to a further increase in negative space charge density. The increase in voltage allows for a large ionization of C_N_ producing a high negative space charge density reduce the current.

**Figure 6:**
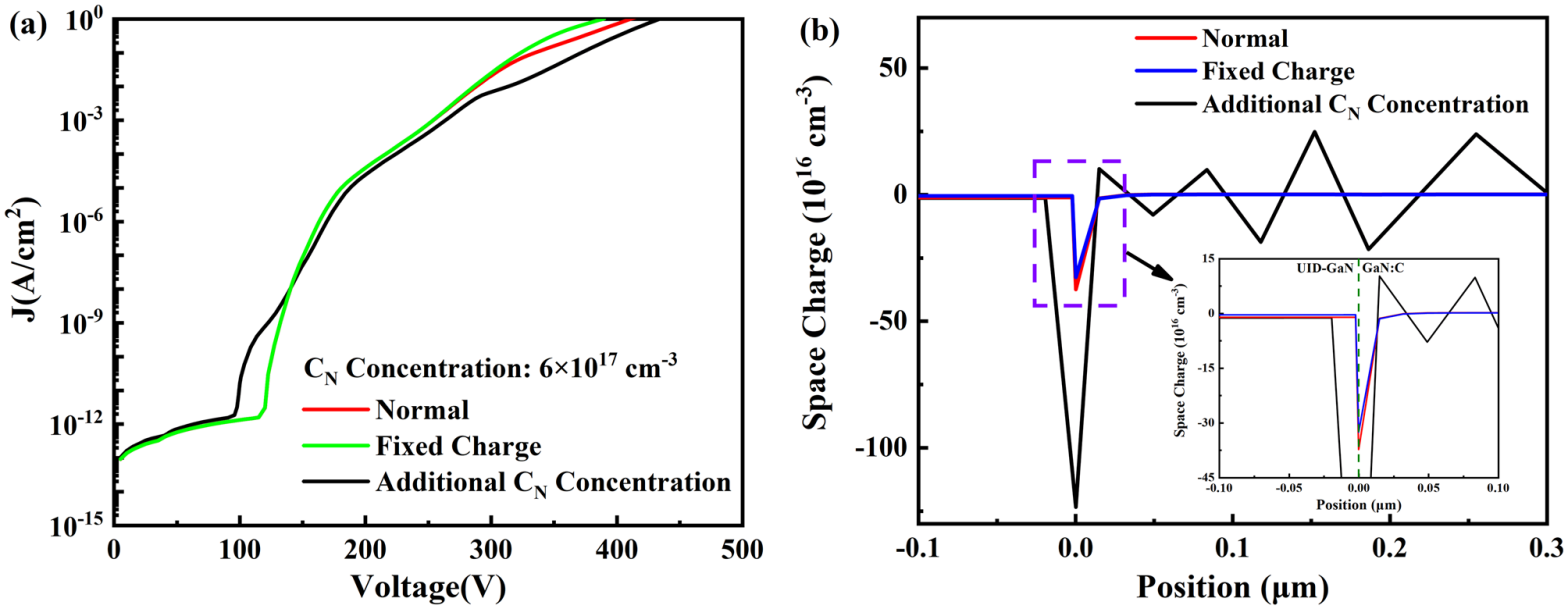
(**a**) Log J-V curve at C_N_ 6 × 10^17^ cm^−3^ without any change (Normal), with the fixed charge at Al_0.25_Ga_0.75_N barrier/UID-GaN interface (Fixed Charge) and with more carbon defects concentration at the top of GaN: C layer (Addition C_N_ Concentration). (**b**) Space charge diagram at breakdown voltages at C_N_ 6 × 10^17^ cm^−3^ without any change (Normal), with the fixed charge at Al_0.25_Ga_0.75_N barrier/UID-GaN interface (Fixed Charge) and with more carbon defects concentration at the top of GaN: C layer (Addition C_N_ Concentration).

To further verify the blocking effect of negative space charge on electron conduction near breakdown at UID-GaN/GaN:C interface, two ways below are used as follow: (1) By introducing an additional 6 × 10^18^ cm^−3^ of C_N_ within the top 0.4 μm of GaN:C layer with C_N_ 6 × 10^17^ cm^−3^ in GaN:C layer to form a stronger positive space charge region in the top of GaN:C layer, thus inducing a stronger negative space charge at UID-GaN/GaN:C interface to enhancing the blocking of electron injection. (2) By introducing 1 × 10^13^ cm^−2^ fixed charge at the Al_0.25_Ga_0.75_N barrier/UID-GaN interface, thereby reducing the negative space charge at UID-GaN/GaN:C interface, thus weakening the blocking of electron injection. The fixed charge value was chosen because it need be greater than the polarized charge at the Al_0.25_Ga_0.75_N barrier/UID-GaN interface for a more significant effect to be possible. The results are shown in Fig. 6. As a result of Fig. 6, the reasons that the negative space charge at the UID-GaN/GaN:C interface block electron conduction and plays an important role in increasing the breakdown voltage, based on the following:

(i) In Fig. 6a, the additional introduction of C_N_ increases the breakdown voltage from 388 to 433 V. For Fig. 6b, the additional introduction of C_N_ in GaN:C significantly increases the negative space charge intensity at the UID-GaN/GaN:C interface. Except for the UID-GaN/GaN:C interface and the additional introduction of C_N_ region. No significant change in space charge in other regions.
(ii) In Fig. 6a, the introduction of fixed charge causes the breakdown voltage to drop from 410 to 388 V and no drop in slope of log J-V at 330 V. There is no significant change in the log J-V curve from 0 to 350 V. In Fig. 6b, comparing normal with fixed charge, the negative space charge intensity at the UID-GaN/GaN:C interface is reduced. For the space charge at UID-GaN/GaN:C interface with the additional C_N_ concentration, the value of space charge is about five times higher than without additional defects.
(iii) The electric field will be more concentrated in regions with a high number of space charges. Therefore, the introduction of additional C_N_ in GaN:C led to much higher space charge, which cause more the electric field to be concentrated in and adjacent to the region where the additional C_N_ is introduced.
(iv) Studies have shown that there is an accumulation of negative space charge at the top of GaN:C and discuss the possibilities for blocking conduction^4, 14^. Würflfl et al. achieved a balance between breakdown voltage and dynamic resistance by additional doping of the top of GaN:C^28^. Our simulations confirm the presence of a strong negative space charge at UID-GaN/GaN:C interface and explain the reason for the additional doping to increase the breakdown voltage in terms of space charge. This shows that our results consistent with reality.

Briefly, for breakdown, the ionization of C_N_ with C_Ga_ at higher electrical stresses results in high negative space charge density at the UID-GaN/GaN:C interface. The negative space charge at the UID-GaN/GaN:C interface blocks the conduction of electrons. Therefore, the increase in C_N_ concentration contributes to the increase in breakdown voltage.

## Conclusion

In this paper, the variation of space charge and the C_N_ and C_Ga_ charging/discharging process from 0 to 448 V in GaN-on-Si epitaxial layers have been investigated. The results indicate that C_N_ in GaN:C layer not only captures electrons but also releases electrons in response to the formation of adjacent space charges. By introducing 1 × 10^13^ cm^−2^ fixed charge at Al_0.25_Ga_0.75_N barrier/UID-GaN interface and additional 6 × 10^18^ cm^−3^ C_N_ concentration in the top 0.4 μm of GaN:C layer, it is confirmed that the blocking of electron injection by negative space charge at UID-GaN/GaN:C interface is the reason for the increase of breakdown voltage. The additional introduction of C_N_ defects can bring the space charge at the UID-GaN/GaN:C interface up to about five times higher than normal case. In the whole process, positive and negative space charges are formed at different positions in GaN: C layer by C_N_ and C_Ga_ charging/discharging process. The study shows that the plateau current and the breakdown voltage can be regulated by utilizing the capture and release behavior of C_N_ at different positions in GaN:C layer. Studying the charging/discharging process of C_N_ and C_Ga_ at different concentrations will help us guide better control of the leakage/breakdown in GaN-on-Si device.

## Data Availability

The datasets used during the current study available from the corresponding author on request.

## References

[1] Amano H (2018). The 2018 GaN power electronics roadmap. J. Phys. D: Appl. Phys..

[2] Tan AK, Hamzah NA, Ahmad MA, Ng SS, Hassan Z (2022). Recent advances and challenges in the MOCVD growth of indium gallium nitride: A brief review. Mater. Sci. Semicond. Process..

[3] Lesnik A (2017). Properties of C-doped GaN: Properties of C-doped GaN. Phys. Status Solidi B.

[4] Uren MJ, Kuball M (2021). Impact of carbon in the buffer on power switching GaN-on-Si and RF GaN-on-SiC HEMTs. Jpn. J. Appl. Phys..

[5] Wu S (2018). Unambiguous identification of carbon location on the N site in semi-insulating GaN. Phys. Rev. Lett..

[6] Mittereder JA (2003). Current collapse induced in AlGaN/GaN high-electron-mobility transistors by bias stress. Appl. Phys. Lett..

[7] Klein PB (2001). Current collapse and the role of carbon in AlGaN/GaN high electron mobility transistors grown by metalorganic vapor-phase epitaxy. Appl. Phys. Lett..

[8] Zhong Y (2022). A review on the GaN-on-Si power electronic devices. Fund. Res..

[9] Huang H (2020). Investigation of carrier compensation traps in n^**−**^ -GaN drift layer by high-temperature deep-level transient spectroscopy. Appl. Phys. Lett..

[10] Lyons JL, Wickramaratne D, Van de Walle CG (2021). A first-principles understanding of point defects and impurities in GaN. J. Appl. Phys..

[11] Zagni N, Chini A, Puglisi FM, Pavan P, Verzellesi G (2021). On the modeling of the donor/acceptor compensation ratio in carbon-doped GaN to univocally reproduce breakdown voltage and current collapse in lateral GaN power HEMTs. Micromachines.

[12] Koller C, Lymperakis L, Pogany D, Pobegen G, Ostermaier C (2021). Mechanism leading to semi-insulating property of carbon-doped GaN: Analysis of donor acceptor ratio and method for its determination. J. Appl. Phys..

[13] Uren MJ (2017). “Leaky dielectric” model for the suppression of dynamic R_on_ in carbon-doped AlGaN/GaN HEMTs. IEEE Trans. Electron Devices.

[14] Yang S, Han S, Sheng K, Chen KJ (2019). Dynamic on-resistance in GaN power devices mechanisms, characterizations, and modeling. IEEE J. Emerg. Sel. Topics Power Electron..

[15] Pooth A, Uren MJ, Cäsar M, Martin T, Kuball M (2015). Charge movement in a GaN-based hetero-structure field effect transistor structure with carbon doped buffer under applied substrate bias. J. Appl. Phys..

[16] Sayadi L (2018). The role of silicon substrate on the leakage current through GaN-on-Si epitaxial layers. IEEE Trans. Electron Devices.

[17] Zhang H (2021). Investigation on dynamic characteristics of AlGaN/GaN lateral schottky barrier diode. Micromachines.

[18] Longobardi, G. *et al.* On the vertical leakage of GaN-on-Si lateral transistors and the effect of emission and trap-to- trap-tunneling through the AlN/Si barrier. in *2017 29th International Symposium on Power Semiconductor Devices and IC's (ISPSD),* 227–230 (IEEE, 2017). doi: 10.23919/ISPSD.2017.7988918.

[19] Yacoub H (2017). Effect of different carbon doping techniques on the dynamic properties of GaN-on-Si buffers. IEEE Trans. Electron Devices.

[20] Uren MJ, Moreke J, Kuball M (2012). Buffer design to minimize current collapse in GaN/AlGaN HFETs. IEEE Trans. Electron Devices.

[21] Cornigli D (2016). TCAD analysis of the leakage current and breakdown versus temperature of GaN-on-Silicon vertical structures. Solid-State Electron..

[22] Zagni N (2022). Experimental and numerical investigation of Poole-Frenkel effect on dynamic R _ON_ transients in C-doped p-GaN HEMTs. Semicond. Sci. Technol..

[23] Song C (2020). The effect of kink and vertical leakage mechanisms in GaN-on-Si epitaxial layers. Semicond. Sci. Technol..

[24] Li X (2018). Investigation on carrier transport through ALN nucleation layer from differently doped Si(111) substrates. IEEE Trans. Electron Devices.

[25] Longobardi G (2019). Suppression technique of vertical leakage current in GaN-on-Si power transistors. Jpn. J. Appl. Phys..

[26] Yacoub H, Fahle D, Finken M, Hahn H, Blumberg C, Prost W, Kalisch H, Heuken M, Vescan A (2014). The effect of the inversion channel at the AlN/Si interface on the vertical breakdown characteristics of GaN-based devices. Semicond. Sci. Technol..

[27] Zhou C, Jiang Q, Huang S, Chen KJ (2012). Vertical leakage/breakdown mechanisms in AlGaN/GaN-on-Si devices. IEEE Electron Device Lett..

[28] Wurfl, J. *et al.* Techniques towards GaN power transistors with improved high voltage dynamic switching properties. in *2013 IEEE International Electron Devices Meeting* 6.1.1–6.1.4 (IEEE, 2013). doi:10.1109/IEDM.2013.6724571.

