# Author Correction: Numerical simulation analysis of carbon defects in the buffer on vertical leakage and breakdown of GaN on silicon epitaxial layers

**DOI:** 10.1038/s41598-024-60017-6

**Published:** 2024-04-23

**Authors:** Weicheng Cao, Chunyan Song, Hui Liao, Ningxuan Yang, Rui Wang, Guanghui Tang, Hongyu Ji

**Affiliations:** 1 Department of Physics, College of Sciences, Shihezi University, Shihezi, 832000 China; 2 Xinjiang Production & Construction Corps Key Laboratory of Advanced Energy Storage Materials and Technology, Shihezi University, Shihezi, 832000 China

Correction to: *Scientific Reports* 10.1038/s41598-023-41678-1, published online 08 September 2023

The original version of this Article contained errors in Figure 2, where the depicted C_N_ Concentration range was incorrect. The original Figure 2 and accompanying legend appear below.

**Figure 2:**
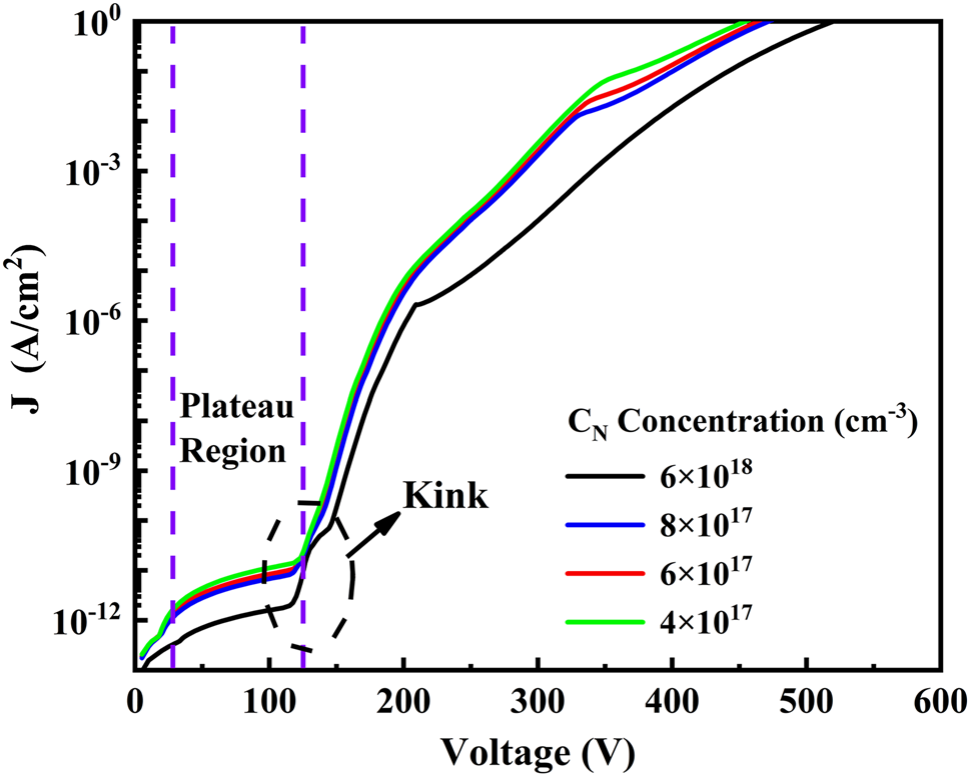
The log J-V curve with the concentration of C_N_ from 6 × 10^16^ cm^−3^, 4 × 10^17^ cm^−3^, 6 × 10^17^ cm^−3^ and 6 × 10^18^ cm^−3^, the concentration of C_Ga_ is 50% of each C_N_ above in GaN:C layer.

The original Article has been corrected.

